# Evidence for the Primo Vascular System above the Epicardia of Rat Hearts

**DOI:** 10.1155/2013/510461

**Published:** 2013-08-20

**Authors:** Ho-Sung Lee, Jeong Yim Lee, Dae-In Kang, Se Hoon Kim, Inhyung Lee, Sang Hyun Park, Seung Zhoo Yoon, Yeon Hee Ryu, Byung-Cheon Lee

**Affiliations:** ^1^Ki Primo Research Laboratory, Division of Electrical Engineering, KAIST Institute for Information Technology Convergence, Korea Advanced Institute of Science and Technology (KAIST), Daejeon 305-701, Republic of Korea; ^2^Pharmacopuncture Medical Research Center, Korean Pharmacopuncture Institute, Seoul 157-200, Republic of Korea; ^3^Department of Pathology, Yonsei University College of Medicine, Seoul 120-752, Republic of Korea; ^4^Department of Veterinary, Seoul National University, Seoul 151-742, Republic of Korea; ^5^Department of Anesthesiology and Pain Medicine, College of Medicine, Korea University, Seoul 136-713, Republic of Korea; ^6^Acupuncture, Moxibustion & Meridian Research Group, Medical Research Division, Korea Institute of Oriental Medicine, Daejeon 305-811, Republic of Korea

## Abstract

We for the first time reported evidence for the existence of a novel network, a PVS, abovethe epicardium of the rat heart. (1) We were consecutively able to visualize the PVs and the PNs above the epicardial spaces of five rats' hearts by using Cr-Hx spraying or injection. (2) Hematoxylin and eosin (H&E) and toluidine blue staining of the PVs and the PNs showed that they consisted of a basophilic matrix; specifically the PNs contained several mast cells, some of which were degranulating into pericardial space. Also, 4′, 6-diamidino-2 phenylindole (DAPI) images of the PVs and the PNs showed that they contained various kinds of cells. (3) Transmission electron microscopic (TEM) longitudinal image of the PVs showed that the sinuses contained many granules with high-electron-density cores in parallel with putative endothelial cells. (4) TEM images of the PNs demonstrated that they consisted of lumen-containing cells surrounded by fibers and that they had mast cells that were degranulating toward the epicardium of the rat heart. The above data suggest that mast-cells-containing novel network exists above the epicardium of the rat heart.

## 1. Introduction

Mast cells have been considered as mediators for allergy reactions because they release histamine [1]. These big-sized cells were first reported as Ehrlich [2]. Histological works have shown these cells to be distributed in various tissues such as skin [3–6], and interestingly there has been an insistence that these mast cells are found at acupuncture points [7–9]. Moreover, oriental medical doctors have suggested that acupuncture effects may be mediated via these mast cells [10].

The concept of the acupuncture meridian system has had a long history that supports Chinese medicine and the clinical effects of acupuncture. However, even in modern medicine, the concept of the acupuncture meridian includes ambiguous ideas, such as nerve system mediation [11–13] or connective tissues [14–16]. A novel concept for the acupuncture meridian system was proposed by Bonghan Kim who demonstrated anatomical realities, Bonghan corpuscles (primo nodes (PNs)), and Bonghan ducts (primo vessels (PVs)), corresponding to the acupuncture meridian system [17].

In previous works on primo nodes and primo vessels, we first reported the existence of mast cells in primo nodes by using transmission electron microscopy [18]; our observations were confirmed by Kwon et al. with more detailed evidence [19]. Interestingly, some research showed that mast cells resided in the connective tissues of hearts [20]. Moreover, the mast cells inside the heart were thought to have specific functions as they contained renin and stem-cell-related factors [21, 22].

Here, we for the first time report our findings on a novel network system above the rat heart by using chromium-hematoxylin staining. We also use light and electron microscope images to demonstrate that this system of primo vessels and primo nodes contains mast cells. In the discussion, we shall share recent findings on mast cells in the heart and in the acupuncture meridian system.

## 2. Material and Method

### 2.1. Laboratory Animal Preparation

Male Wistar rats aged 5~6 weeks (*n* = 7; Samtako Bio Korea, Bio Korea, Gyunggi-Do, Korea) were housed in a room that was temperature controlled at 24~25°C and light controlled with a 12/12-hour light/dark cycle and were provided water and commercial rat chow ad libitum. The rats were acclimatized for 1 week before the experiment. These experiments were carried out in accordance with the guidelines (KAIST approval number: KA2011-13) of the Laboratory Animal Care Advisory Committee of the Korea Advanced Institute of Science and Technology (KAIST). The rats were anesthetized by using an intramuscular injection of a combination of ketamine (45 mg/kg) and lompun (5 mg/kg) into the right hind femoral limb.

### 2.2. Preparation of Chromium-Hematoxylin Solution (Cr-Hx)

Fifty ml of hematoxylin (1%) and 50 ml of chromium potassium sulfate (3%) were mixed to make 100 ml of a Cr-Hx solution to which 0.1 g of potassium iodate had been added. The solution was boiled until it became a deep blue. The deep-blue solution was filtered with a 0.45 *μ*m pore size membrane filter before use. For the visualizing experiment, we diluted Cr-Hx by a factor of 10 with phosphate buffered saline (PBS, pH 7.4).

### 2.3. Surgical and Observation Procedures

In order to visualize the network of the primo vascular system (PVS) that consists of primo nodes (PNs) and primo vessels (PVs), we used different staining methods. One method was to spray 10% Cr-Hx solution in phosphate-buffered saline at pH 7.4 (PBS) onto the surfaces of the epicardia of the rat hearts after the chests had been opened under deep anesthesia. In the other method, which was better for visualizing the primo vascular system above the epicardium of the rat heart, we opened the abdominal cavity to find the diaphragm and injected about 0.5 ml of 10% Cr-Hx solution in PBS into the pericardial cavity from the opposite side of diaphragm for 30 minutes to 2 hours. However, because the Cr-Hx solution is injected manually, the injection point may differ slightly from injection to injection. In order to overcome this shortcoming, we confirmed where the Cr-Hx solution had been injected by dissecting the chest under a stereomicroscope (SZ61, Olympus, Japan). After the chest had been opened, we exposed the transparent pericardium and visualized the PVS in the pericardial cavity; then, we cut the pericardium and washed it with PBS to clean out the Cr-Hx solution remaining in the epicardium of the heart. The washing step was performed under a stereomicroscope during the dissection of the rat heart.

### 2.4. Microscopic Examination

The isolated whole specimens were first stained with 4′,6′-diamidino-2-phenylindole (DAPI) and examined using light microscopy. For the light microscope and the transmission electron microscope examinations of the PV cross sections, the specimens were fixed in 2.5% paraformaldehyde and 2.5% glutaraldehyde in a neutral 0.1 M phosphate buffer for 1 hour. The specimens were postfixed for 1 hour in 1% (w/v) osmic acid dissolved in PBS, dehydrated in graded ethanol, and embedded in Epon812 (EMS, Fort Washington, PA, USA). Semithin sections of 1 *μ*m in thickness stained with 1% toluidine blue were observed and photographed under a light microscope (BX 53, Olympus) with a CCD camera (eXcope X3, DIX, Korea). Ultrathin sections were cut and mounted on nickel grids and were double stained with uranyl acetate, followed by staining with lead citrate. The sections were examined with a Technai G2 Spirit transmission electron microscope (FEI, USA). 

## 3. Results 

**Figure 1:**
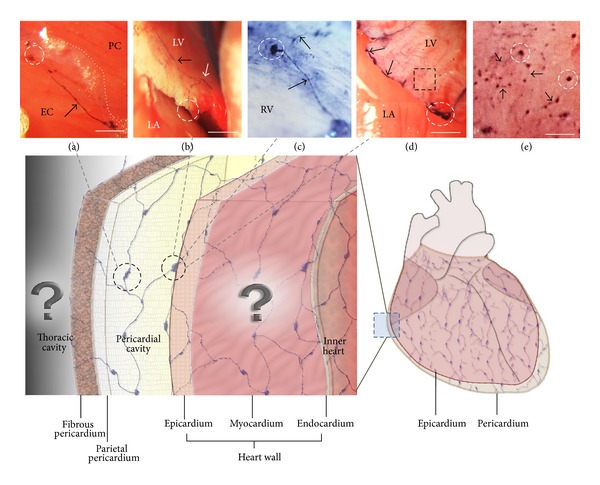
Illustration of the PVS above the epicardium of the rat heart with real stereoscopic images of the PVS stained with Cr-Hx. The illustration suggests the network system of the PVS; the two question marks remain to be studied. The real stereomicroscopic images in (a), (b), (c), and (d) show PNs (dotted circles) and PVs (arrows). The stereoscopic image in (e) is a magnified image of the area in the dotted rectangle of image (d). Tiny node-like structures are visualized in image (e). The scale bars of (a), (b), (c), (d), and (e) are 507 *μ*m, 1106 *μ*m, 207 *μ*m, 430 *μ*m, and 56 *μ*m, respectively.

**Figure 2:**
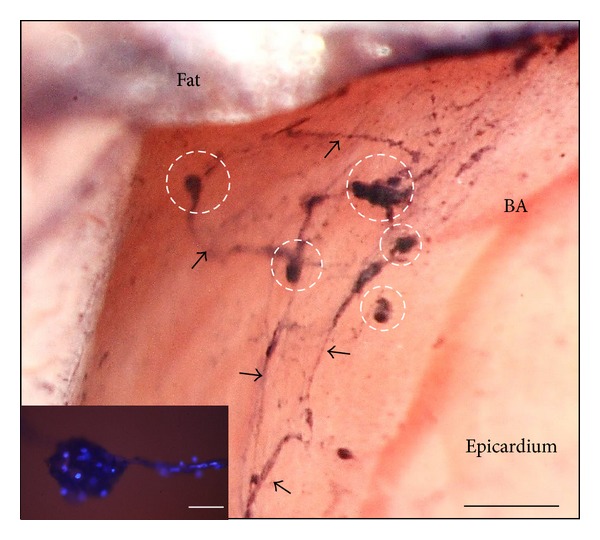
Representative stereoscopic image of the PVS, stained with Cr-Hx, above the epicardium of the rat heart. Dotted circles indicate primo PNs, and arrows mean PVs. BA means branch of artery, which is not stained with Cr-Hx. The inset fluorescence image of PNs and PVs stained with (DAPI) demonstrates that they have several kinds of cells. The scale bars of the main figure and the inset are 270 *μ*m and 40 *μ*m, respectively.

As recorded in Table 1, we consistently were able to visualize the PVs and the PNs above the epicardia of five rats' hearts. The PNs were oval shaped, and the PVs looked threadlike with diameters of about 15 *μ*m. Some stereomicroscopic images of PVs and PNs visualized by using Cr-Hx staining, along with an illustration of the heart, are shown in Figure 1. Figure 1 shows distinctive images of PNs and PVs; specifically, a fine network view of a PV is demonstrated under high magnification in image (e). A representative magnified image is also shown in Figure 2, which shows PNs and PVs stained with DAPI and demonstrates the distinctive network of the PV. We took one PN with a PV to reveal the pattern of nuclei.

**Figure 3:**
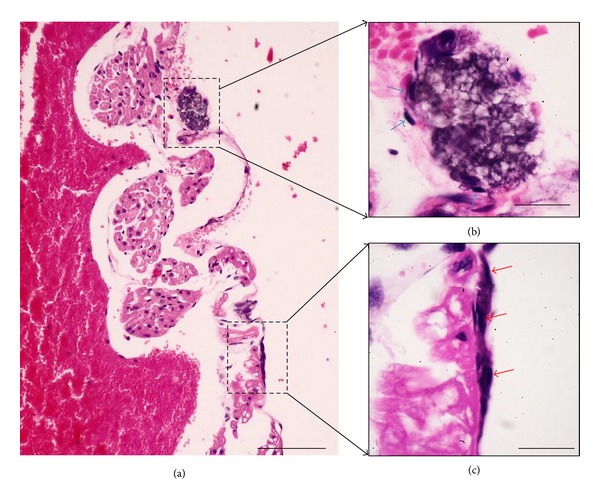
(a) Low-magnification image showing two basophilic structures located above the eosinophilic epicardium of the rat heart. The two structures are magnified into (b) and (c), respectively. (b) Sectioned PN enveloped by heavily hematoxylin-and-eosin-stained cells (arrows). (c) Longitudinal-sectioned PV also consisting of strongly hematoxylin-and-eosin-stained cells (arrows). The scale bars of (a), (b), and (c) are 85 *μ*m, 15 *μ*m, and 18 *μ*m, respectively.

**Figure 4:**
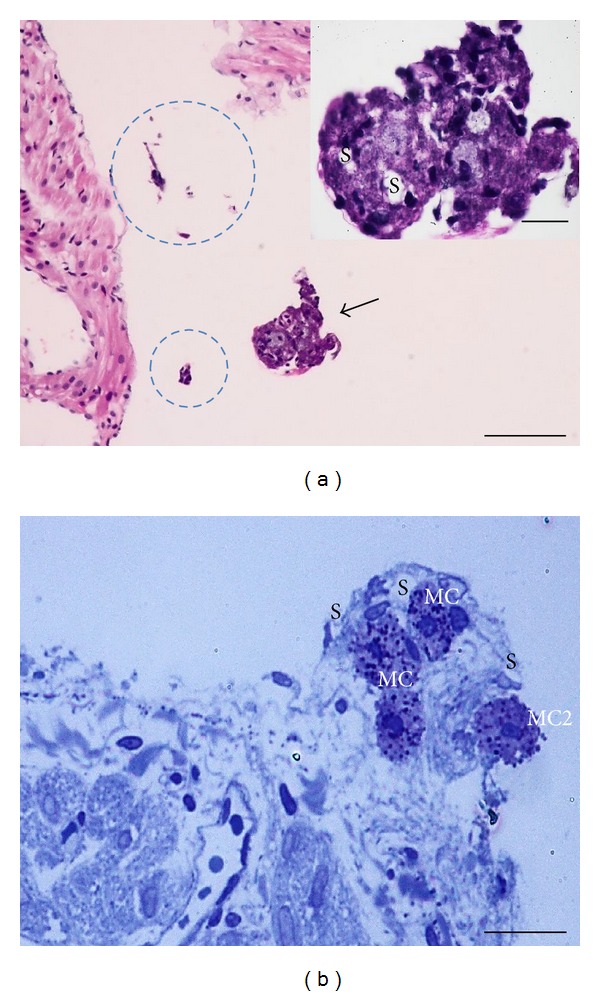
Comparative positions of a PN and a PV stained with (a) hematoxylin and eosin (H&E) and with (b) toluidine blue (TB). Image (a) shows a randomly cut PV (dotted circles) and a cross-sectioned PN (arrow) above the epicardium (EC). H&E staining of the PV and the PN suggest they are basophilic; however, the epicardium is eosinophilic. The inset image is a magnified view of a PN with sinuses (S). Image (b) shows a toluidine-blue-stained PN in which four mast cells (MC) are found. Among the MCs, one MC is just releasing from the PN. The scale bars of (a), (a)'s inset, and (b) are 30 *μ*m, 12 *μ*m, and 25 *μ*m, respectively.

In order to investigate cross-sectional images of PNs and PVs, we sectioned and stained them with hematoxylin and eosin (H&E).  Figure 3 presents representative hematoxylin-and-eosin-stained images of PNs and PVs above the epicardium of the rat heart. The PNs and the PVs are basophilic; however, the epicardium-containing heart tissues are eosinophilic. For more information on the relative positions of the epicardium and the PVS, as shown in Figure 4, we used two images. One image was H&E-stained PVs and PNs positioned apart from the epicardium. The other involved toluidine-blue-stained PNs almost embedded in the epicardium of the rat heart. Noticeably, the PN contained mast cells, among which a mast cell was observed to be degranulating into the pericardium of the rat heart.

**Figure 5:**
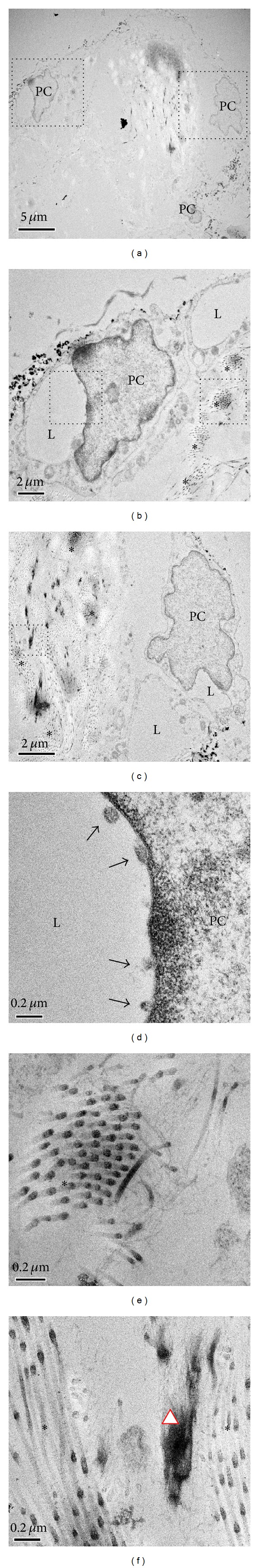
Transmission electron microscopy images of cross-sectioned PNs. (a) Low-magnification view of a PN in which the outermost area consists of two cells (PC: putative endothelial cells). (b) Magnified view of the left rectangle in image (a), showing distinctive lumens (L) in the PC. (c) Magnified view of the right rectangle in image (a), also showing a lumen-containing PC. (d) High-magnification view of the lumen (L) indicated by the rectangle in (b); in (d) some microgranules are seen (arrows). Asterisks in images (b) and (c) are magnified into images (e) and (f), respectively, and demonstrate bundles of collagen fibers. The triangle in image (f) means cluster of fine fibers.

**Figure 6:**
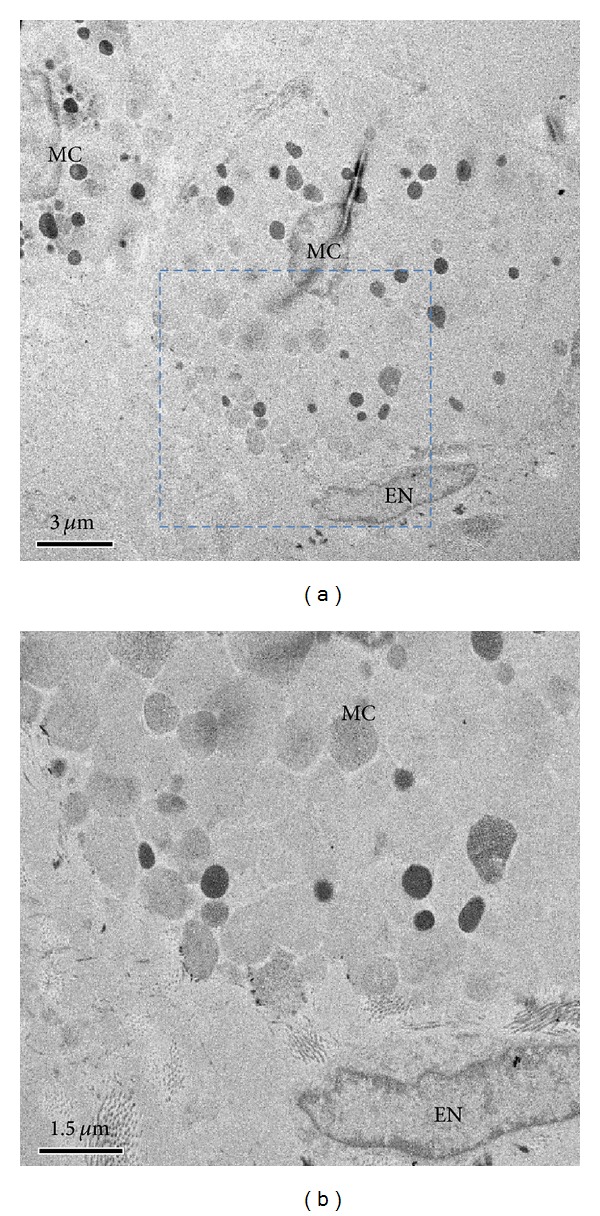
Transmission electron microscopy images of mast cells in cross-sectioned PNs. (a) Low-magnification view of two mast cells just above the EN of the heart. (b) Magnified view of the rectangle in image (a) shows more distinctive granules just above the EN.

**Figure 7:**
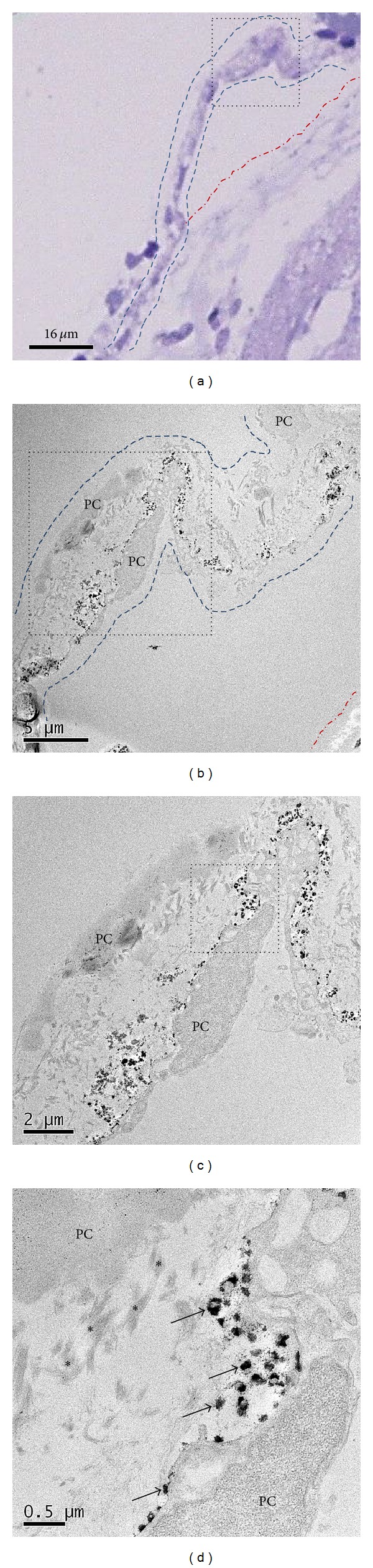
Light and transmission electron microscopy (TEM) images of a longitudinal sectioned PV just above the epicardium. Image (a) shows a toluidine blue-stained PV (dotted lined) above the epicardium (red-colored line). (b) TEM low-magnification view of longitudinal sectioned PV corresponding to the rectangle of image (a). Red-colored line also indicates the epicardium. Image (c) is magnified from the rectangle of (b). Image (d) shows high magnification of the rectangle of (c) in which there are high-electron-density microgranules (arrows) in the lumen of the PV. Asterisks indicate the presence of fine fibers. PC means putative endothelial cells.

For more detailed information on the PNs and the PVs, we conducted transmission electron microscopy (TEM). Figures 5 and 6 demonstrate that PNs consist of two kinds of fibers: one is collagen fibers, and the other is very fine fibers. Also, TEM images of PNs clearly show mast cells' degranulating. As shown in Figure 7, we also found that longitudinal images of PNs showed the presence of high-electron-density microgranules in the lumen of the PV.

## 4. Discussion

The data in this research suggest three characteristics for the newly found network, the primo vascular system, and we observed the following. (1) Hematoxylin-and-eosin (H&E) and toluidine-blue staining of the PVs and the PNs showed that they consisted of a basophilic matrix; specifically, a PN was observed to contain several mast cells, one of which was degranulating into the pericardial space. The DAPI-stained PVs and PNs showed that they contained some cells. (2) Transmission-electron-microscope (TEM) longitudinal image of the PVs showed that the lumen contained many granules with high-electron-density cores in parallel with putative endothelial cells. (3) A TEM image of a PN demonstrated that it consisted of lumen-containing cells surrounded by fibers and that it had mast cells degranulating toward the epicardium of the rat heart. Given all the experimental data, we draw a tentative conclusion that a novel system exists under the pericardial space of the rat heart.

Kim was the first to discover a network floating freely above internal organs such as the heart [17]. He suggested the following peculiar characteristic of the network system (the primo vascular system): it contained some flowing DNA that was different from nuclei DNA and extracellular DNA (eDNA). Thus, the strong basophilic matrix that we observed could be interpreted as supporting his eDNA concept. In a previous work, we were able to demonstrate the existence of eDNA in the PVS above the pia mater of the rat brain [23], and recently, we were able to detect DNA signals in primo vessels floating in the lymphatic vessels of rabbits [24]. Therefore, one of authors, Lee BC, presented a hypothesis for an overall feature of the PVS; that is, the PVS is an eDNA circulation system for the acupuncture meridian system [25, 26].

Another interestingly finding is the existence of mast cells in PNs. Recently, the importance of mast cells emerged after reports that mast cells contained renin and stem-cell-related factors [1, 20, 27, 28]. This research shows the importance of mast cells in terms of renin-containing cells because renin has been considered to be a key blood-pressure-modulating hormone and because the newly found network is located under the pericardial space of the rat heart [22, 29]. Recently, cardiologists have paid attention to the existence of mast cells in connective tissues inside the heart as renin is mainly found only in the kidney as a renin-angiotensin system [21]. However, the PNs in the newly found network contained several mast cells, some of which were observed to be degranulating into not only the pericardial space but also the epicardium of the rat heart. Thus, our data on mast cells suggest that the primo network above the epicardium of the rat heart may have a role in modulating blood pressure directly. 

On the other hand, acupuncture points have been reported to consist of more mast cells than nonacupuncture points. This finding encouraged oriental medical doctors to connect mast cells to the function of acupuncture. Many clinical and research data reported that the stimulation of acupuncture points modulated blood pressure [30]. All of the above data and ideas imply that newly found PVS above the heart plays a role in transmitting certain signals to modulate blood pressure.

During examination of TEM images, we noticed that the PN had sinus-containing cells; moreover, the PN connected with the PV clearly showed high-electron-density microvesicles aligned in the lumen structure along adjacent cells. Noticeably, these microvesicles in the PN and the PV share characteristics similar to those of general endothelial cells in terms of the TEM image. Based on our analysis of the TEM images, we temporarily conjecture that the PVS found above the heart may be endothelial cells with fluid channels. Precisely, we examined the microvesicles found in the PV and found them to be very similar to neuroendocrine microvesicles in terms of high electron density, size, and overall morphology [31]. This conjecture comes from Bonghan theory [17], our previous hormone analysis [32], and the characteristics of Cr-Hx staining, which was used to visualize the PVS [33].

Thus, given these TEM data, we paid attention to Bonghan theory that the PVS functions as a hormone-transporting channel. The data presented here and in previous works provide evidence that supports Bonghan Kim's first insistence that the PVS plays a role in transporting hormones independently from the blood stream. As Bonghan Kim considered the PVS to be an anatomical acupuncture meridian system, our findings, as well as those of others, should encourage both western cardiologists and oriental medical doctors to investigate the PVS above the heart in terms of both cardiology and a heart-related meridian system.

## Figures and Tables

**Table 1:** Data on primo nodes and primo vessels visualized above epicardium of rat heart. The numbers are rat numbers sacrificed for these experiments which were all successfully visualized. *L* means the longest and shortest length of oval shaped primo nodes. *D* and *d* indicate the thickest and thinnest diameter of primo vessels visualized in each rat. The methods applied were spray or injection method.

Primo nodes and primo vessels above epicardium of heart of rat				
Number	*L* (mm × mm)	*D* (μm)	*d* (μm)	Method
1	0.15 × 0.07	29	10	Spray
	0.09 × 0.07	22	8	Spray

2	0.23 × 0.16	28	10	Spray
	0.14 × 0.07	19	12	Spray
	0.16 × 0.09	27	15	Spray
	0.09 × 0.06	27	17	Spray

3	0.13 × 0.13	17	5	Injection
	0.56 × 0.19	22	14	Injection
	0.20 × 0.42	22	14	Injection
	0.25 × 0.18	22	14	Injection

4	0.20 × 0.41	33	24	Injection
	0.24 × 0.11	38	26	Injection
	0.33 × 0.11	20	11	Injection

5	0.08 × 0.04	13	4	Injection
	0.06 × 0.04	27	9	Injection
	0.07 × 0.02	9	6	Injection
	0.17 × 0.13	17	4	Injection
	0.07 × 0.05	11	7	Injection
	0.12 × 0.03	14	4	Injection
	0.07 × 0.03	6	4	Injection

Average ± SD	0.17 ± 0.12 × 0.12 ± 0.11	21.15 ± 8.20	10.90 ± 6.33	
